# CXCL10 Gene Promoter Polymorphism -1447A>G Correlates with Plasma CXCL10 Levels and is Associated with Male Susceptibility to Cerebral Malaria

**DOI:** 10.1371/journal.pone.0081329

**Published:** 2013-12-09

**Authors:** Nana Wilson, Adel Driss, Wesley Solomon, Carmen Dickinson-Copeland, Hassana Salifu, Vidhan Jain, Neeru Singh, Jonathan Stiles

**Affiliations:** 1 Department of Microbiology, Biochemistry and Immunology, Morehouse School of Medicine, Atlanta, Georgia, United States of America; 2 Regional Medical Research Center for Tribals (ICMR), Nagpur Road, Garha, Jabalpur, Madhya Pradesh, India; 3 National Institute of Malaria Research Field Unit (ICMR), Jabalpur, Madhya Pradesh, India; Albert Einstein Institute for Research and Education, Brazil

## Abstract

The risk factors for cerebral malaria (CM) and the wide variation in clinical manifestations of malaria are poorly understood. Recent studies indicate that interferon gamma inducible chemokine, CXCL10, is a strong predictor of both human and experimental cerebral malaria. Increased plasma and cerebrospinal fluid levels of CXCL10 were tightly associated with fatal CM in Indian and Ghanaian patients. In the present study, we hypothesized that in a subset of malaria patients, CM susceptibility is associated with variation in CXCL10 expression. We determined whether polymorphisms in the CXCL10 gene promoter region played a role in the clinical status of malaria patients and addressed the genetic basis of CXCL10 expression during malaria infection. Following extensive bioinformatics analyses, two reported single nucleotide polymorphisms in the CXCL10 promoter (−135G>A [rs56061981] and −1447A>G [rs4508917]) were identified among 66 CM and 69 non-CM Indian patients using PCR-restriction fragment length polymorphism assay. Individuals with the −1447(A/G) genotype were susceptible to CM (adjusted odds ratio [AOR] = 2.60, 95% CI = 1.51–5.85, p = 0.021). In addition, individuals with the −1447(A/G) genotype had significantly higher plasma CXCL10 levels than individuals with the −1447(A/A) genotype. Stratifying patients according to gender, the observed association of CM with over expression of CXCL10 were more pronounced in males than in female patients (AOR = 5.47, 95% CI = 1.34–22.29, p = 0.018). Furthermore, −135G>A polymorphism conferred a decreased risk of CM among males (AOR = 0.19, 95% CI = 0.05–0.78, p = 0.021). Polymorphisms in the CXCL10 gene promoter sequence were associated with increased CXCL10 production, which is linked to severity of CM. These results suggest that the −1447A>G polymorphism in CXCL10 gene promoter could be partly responsible for the reported variation underlying severity of CM outcomes particularly in males.

## Introduction

Malaria remains a major cause of global morbidity and mortality. Globally, an estimated 219 million cases of malaria were reported in 2010 resulting in 660,000 deaths [1]. Cerebral malaria (CM) is a central nervous system complication of *Plasmodium falciparum* infection and accounts for about 80% of fatal malaria cases [2]. Although mortality is unacceptably high, about 20% of malaria cases develop into CM [3]–[5] suggesting that CM is a sub-population-specific targeted syndrome [5]. However, the mechanism(s) modulating CM progression in malaria patients is unclear. Several factors have been implicated in the development of CM including host and parasite genetic factors considered as major contributors [5]. The risk factors for CM and the wide variation in clinical manifestations of malaria are poorly understood [6]. Variations in disease severity and syndromic phenotype constitute a major challenge to our understanding of the disease, its treatment, and control [6].

Pro-inflammatory cytokines and chemokines have been implicated in CM but none of these molecules has been identified as a key regulator in all settings [7]. In CM, TNF-α and other cytokines such as IFN-γ contribute to pathogenesis of the disease by up-regulating the expression of adhesion molecules such as ICAM-1 to bind parasitized red blood cells to the vascular endothelium [8]. The binding of parasitized red blood cells by the adhesion molecules to the cerebral endothelium contributes to modifications of blood-brain barrier integrity thereby playing a crucial role in pathogenesis of CM [9]. However, in a clinical trial, treatment with monoclonal anti-TNF-α antibody did not protect against CM and exacerbated neurological sequelae [10].

CXCL10 is a chemokine induced by IFN-γ and TNF-α that is involved in the regulation of key biological responses including inflammation, chemotaxis, and angiogenesis [2], [11]. Several studies have focused on defining the role of CXCL10 in CM. Increased CXCL10 mRNA expression has been reported in the brain of *P. berghei* ANKA infected mice with experimental CM (ECM) [12]–[14]. Furthermore, CXCL10 is the earliest chemokine to be up-regulated in the brain of mice with ECM and occurs prior to T-cell infiltration [12]. In a recent study of Ghanaian children with CM, CXCL10 was elevated in the cerebrospinal fluid (CSF) as well as serum and was associated with CM mortality [15]. Similarly, elevated plasma levels of CXCL10 were observed in Indian CM patients and were associated with mortality [16], [17]. Furthermore, CXCL10 has been shown to be a predictor of CM survival and/or fatal CM [17]. These results strongly suggest that up-regulation of CXCL10 is an important early event in CM pathogenesis that precedes the clinical manifestation of the disease and subsequent exacerbation of the syndrome. From these observations, it is conceivable that genetic variants of CXCL10 gene products and associated sequences could confer increased susceptibility to severe forms of CM, while influencing serum/plasma levels of the protein. Increased CXCL10 production induces brain vascular endothelial and glia cell apoptosis resulting in blood-brain barrier dysfunction and damage during CM pathogenesis [7]. Since both human CM and murine ECM show major involvement of CXCL10 in pathogenesis of the disease, it is expected that sequence variations in the CXCL10 gene may result in altered expression of transcriptional and translational products thereby modulating CM progression in malaria patients.

Single nucleotide polymorphisms (SNPs) in the CXCL10 gene have been described to be associated with diabetes [18], hepatitis [19], tuberculosis [20], Alzheimer's disease [21], and multiple sclerosis [22]. One of the SNPs represents a guanine (G) to adenine (A) transition at position −135 (rs56061981) within the CXCL10 gene promoter (−135G>A). This variant has been associated with increased susceptibility to liver disease among hepatitis B virus carriers [19] and conferred protection against tuberculosis in a Chinese cohort [20]. The −135G>A (rs56061981) variant affects CXCL10 promoter activity which contributes to CXCL10 expression via NFκB transactivation [20] indicating that CXCL10 gene polymorphism may be associated with the observed disparities in CXCL10 levels in a subset of individuals. Regulation of the CXCL10 gene ultimately leads to differential expression of CXCL10 protein. In the present study, we hypothesized that CM susceptibility in a subset of malaria patients is genetically linked to differential expression of CXCL10. We assessed the correlation between polymorphisms in the CXCL10 gene promoter and clinical status of malaria patients and determined the genetic basis of CXCL10 production during *P. falciparum* infection in Indian patients with CM compared to non-CM patients. We assessed the role of the polymorphisms within the CXCL10 promoter region in differential expression of plasma CXCL10 and in promoting genetic risk factors that affect the clinical severity of malaria in the population.

## Results

### Patient characteristics

In the present study, blood samples drawn from 135 Indian malaria patients (69 non-CM and 66 CM) were genotyped for CXCL10 promoter polymorphisms at position −135 and −1447. Table 1 provides characteristics of all subjects as well as the frequencies of genotypes and alleles in the CXCL10 promoter polymorphisms. There were no differences in age and gender between non-CM and CM patients (Table 1). Anemia was present in all groups, but the hemoglobin levels were significantly lower in the CM patients when compared to non-CM patients (p<0.001, Table 1). Peripheral blood parasitemia was higher among CM patients compared to non-CM patients (p<0.001, Table 1).

**Table 1:** Clinical and parasitological characteristics of patients.

Characteristics	Non-CM	CM	p-value
	(n = 69)	(n = 66)	
**Gender**			
Male	35 (50.7%)	28 (42.4%)	0.334
Female	34 (49.3%)	38 (57.6%)	
**Median age (IQR)**	26 (16–40)	24 (12–41)	0.425
**Median coma score (IQR)**	14 (14–14)	6 (4–8)	<0.001
**Median parasitemia (IQR)**	613 (43–2080)	1760 (733.5–8720.3)	<0.001
**Median hemoglobin (IQR)**	9 (8–11)	8 (6–10)	<0.001

IQR  =  interquartile range; CM  =  cerebral malaria

### Association of −135G>A and −1447A>G promoter polymorphisms with cerebral malaria

The genotype and allele frequencies of the −135G>A and −1447A>G polymorphisms in the promoter region of CXCL10 gene were studied in a cohort of 135 cases of malaria belonging to the CM and non-CM groups (Table 2). The genotype distribution of the −135G>A polymorphism followed Hardy-Weinberg equilibrium in both non-CM and CM patients. The genotype distribution of the −1447A>G polymorphism was not in Hardy-Weinberg equilibrium in CM patients. However, analysis revealed strong pairwise linkage disequilibrium (D′ = 1) between −135G>A and −1447A>G polymorphisms within the promoter region of CXCL10 gene. Although, there were no significant differences in genotype distribution of −135G>A between non-CM and CM patients, the frequency of CXCL10 -135 variant A allele was found to be 0.217 in non-CM and 0.182 in CM (Table 2). In our study group we did not find individuals with the A/A genotype for −135G>A promoter polymorphism. However, there was a significant difference in the genotype distribution of −1447A>G between non-CM and CM patients (p = 0.033). The CXCL10 −1447G allele frequency was higher among CM patients than among non-CM patients (0.311 vs. 0.246). Among the CM patients we did not find individuals with the G/G genotype for −1447A>G promoter polymorphism.

**Table 2:** Genotype and allele frequency of CXCL10 polymorphisms in patients with cerebral malaria and non-cerebral malaria.

CXCL10	Non-CM	CM	OR (95% CI)	p value
polymorphism	(n = 69)	(n = 66)		
**−135G>A**				
G/G	39 (56.5%)	42 (63.6%)	1.00 (Ref.)	
G/A	30 (43.5%)	24 (33.3%)	0.94 (0.43–2.19)	0.938
A/A	-	-		
A allele	0.217	0.182		
G allele	0.783	0.818		
**−1447A>G**				
A/A	37 (53.6%)	25 (37.9%)	1.00 (Ref.)	
A/G	30 (43.5%)	41 (62.1%)	2.60 (1.51–5.85)	0.021
G/G	2 (2.9%)	-		
A allele	0.754	0.689		
G allele	0.246	0.311		

CM  =  cerebral malaria; OR  =  odds ratio; CI  =  confidence interval

Multivariable logistic regression analysis indicates that adjusting for age, gender, and parasitemia, individuals with heterozygous −1447(A/G) genotype were more susceptible to CM (adjusted OR [AOR] = 2.60; 95% CI = 1.51–5.85; p = 0.021) compared to those with homozygous −1447(A/A) genotype (Table 2). The significant association of −1447G allele in heterozygous individuals with CM when the −1447(A/A) genotype was used as the reference group suggests that the −1447G allele was a putative risk allele in the susceptibility to CM. When both genotypes of CXCL10 polymorphisms were grouped with −135(G/G)/−1447(A/A) genotypes as the reference group (Table 3), a significant increase in risk of CM was observed with −135(G/G)/−1447(A/G) genotypes (AOR = 12.58; 95% CI = 2.69–59.30; p = 0.001).

**Table 3:** Combined genotype frequencies of CXCL10 polymorphisms in patients with cerebral malaria and non-cerebral malaria.

Combined genotype		Non-CM	CM	OR (95% CI)	p value
−135G>A	−1447A>G	(n = 69)	(n = 66)		
G/G	A/A	37 (53.6%)	25 (37.9%)	1.00 (Ref.)	
G/G	A/G	2 (2.9%)	17 (25.8%)	12.58 (2.69−59.30)	0.001
G/A	A/G	28 (40.6%)	24 (36.4%)	1.27 (0.60–2.67)	0.531
G/A	G/G	2 (2.9%)	-		

CM  =  cerebral malaria; OR  =  odds ratio; CI  =  confidence interval

### Stratification analyses of −135G>A and −1447A>G and risk for cerebral malaria

Stratification analyses were done to evaluate the effect of −135G>A and −1447A>G genotypes on CM risk according to gender (Table 4 and 5). In stratification analysis, a significant increased risk for CM associated with the −1447(A/G) genotype was evident among male patients (AOR = 5.47; 95% CI = 1.34–22.29; p = 0.018, Table 4) compared with the −1447(A/A) genotype. However, the association was not significant among female patients (AOR = 1.85; 95% CI = 0.60–5.64; p = 0.282, Table 5). Furthermore, the −135(G/A) genotype appeared as a protective genotype against CM among the male patients (AOR = 0.19; 95% CI = 0.05–0.78; p = 0.021; Table 4) compared with the −135(G/G) genotype. However, the association was not significant in female patients (AOR = 1.48; 95% CI = 0.58–3.77; p = 0.415, Table 5).

**Table 4:** Stratification analysis of male gender on association between CXCL10 promoter polymorphisms (−135G>A and −1447A>G) and cerebral malaria.

CXCL10	Non-CM	CM	OR (95% CI)	p value
polymorphism	(n = 35)	(n = 28)		
**−135G>A**				
G/G	21 (60.0%)	25 (89.3%)	1.00 (Ref.)	
G/A	14 (40.0%)	3 (10.7%)	0.19 (0.05–0.78)	0.021
A/A	-	-		
G allele	0.8	0.946		
A allele	0.2	0.054		
**−1447A>G**				
A/A	19 (54.3%)	8 (28.6%)	1.00 (Ref.)	
A/G	14 (40.0%)	20 (71.4%)	5.47 (1.34–22.29)	0.018
G/G	2 (5.7%)	-		
A allele	0.743	0.643		
G allele	0.257	0.357		

CM  =  cerebral malaria; OR  =  odds ratio; CI  =  confidence interval

**Table 5:** Stratification analysis of female gender on association between CXCL10 promoter polymorphisms (−135G>A and −1447A>G) and cerebral malaria.

CXCL10 polymorphism	Non-CM	CM	OR (95% CI)	p value
	(n = 34)	(n = 38)		
**−135G>A**				
G/G	18 (52.9%)	17 (44.7%)	1.00 (Ref.)	
G/A	16 (47.1%)	21 (55.3%)	1.48 (0.58–3.77)	0.415
A/A	-	-		
G allele	0.765	0.724		
A allele	0.235	0.276		
**−1447A>G**				
A/A	18 (52.9%)	17 (44.7%)	1.00 (Ref.)	
A/G	16 (47.1%)	21 (55.3%)	1.85 (0.60–5.64)	0.282
G/G	-	-		
A allele	0.765	0.724		
G allele	0.235	0.276		

CM  =  cerebral malaria; OR  =  odds ratio; CI  =  confidence interval

When both genotypes of CXCL10 polymorphisms were combined, taking −135(G/G)/−1447(A/A) genotypes as the reference group (Table 6), a significantly increased risk of CM was observed with −135(G/G)/−1447(A/G) genotypes (AOR = 20.38; 95% CI = 3.06–86.84; p<0.0001, Table 6) among male patients. However, in −135(G/A)/−1447(A/G) genotypes, the −135(G/A) genotype appeared to cancel the association of −1447(A/G) genotype with CM in male patients although it was not statistically significant (AOR = 0.79; 95% CI = 0.17–3.78; p = 0.770, Table 6). There were no associations with the combined genotypes of CXCL10 polymorphisms for CM risk among the female patients (Table 6).

**Table 6:** Stratification analysis of combined genotype frequencies of CXCL10 polymorphisms in patients with cerebral malaria and non-cerebral malaria.

Gender	Combined genotype		Non-CM	CM	OR (95% CI)	p value
	−135G>A	−1447A>G				
Male Only			**n = 35**	**n = 28**		
	G/G	A/A	19 (54.3%)	8 (28.6%)	1.00 (Ref.)	
	G/G	A/G	2 (5.7%)	17 (60.7%)	20.38 (3.06–86.84)	<0.0001
	G/A	A/G	12 (34.3%)	3 (10.7%)	0.79 (0.17–3.78)	0.77
	G/A	G/G	2 (5.7%)	-		
						
Female Only			**n = 34**	**n = 38**		
	G/G	A/A	18 (52.9%)	17 (44.7%)	1.00 (Ref.)	
	G/G	A/G	-	-	-	
	G/A	A/G	16 (47.1%)	21 (55.3%)	1.48 (0.58–3.77)	0.415
	G/A	G/G	-	-		

CM  =  cerebral malaria; OR  =  odds ratio; CI  =  confidence interval

### Plasma levels of CXCL10

**Figure 1:**
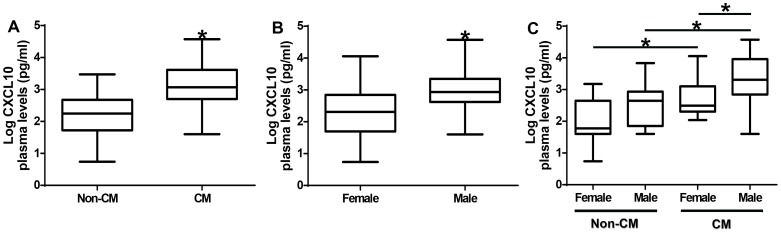
Plasma levels of CXCL10 protein among the study group. (A) Association of plasma CXCL10 protein levels with CM. Cerebral malaria patients have significantly higher plasma CXCL10 levels than non-CM patients (p<0.0001). (B) Association of plasma CXCL10 protein levels with gender. Males have significantly higher plasma CXCL10 levels than females (p<0.0001). (C) Association of plasma CXCL10 protein levels with CM stratified by gender. Both males and females in the CM group have significantly higher plasma CXCL10 levels than the corresponding gender in the non-CM group (p<0.05). Box plot represent log-transformed medians with 25^th^ and 75^th^ percentiles, bars for 10^th^ and 90^th^ percentiles. Significant differences of CXCL10 median levels between the groups were determined by Mann-Whitney U test or Kruskal Wallis test with Dunn's multiple comparisons tests. Asterisk (*) indicates a significant difference (p<0.05). CM  =  cerebral malaria.

We determined the plasma CXCL10 levels among the study participants. Figure 1A shows increased plasma CXCL10 levels in CM patients compared to non-CM patients (p<0.0001). Further, we assessed CXCL10 levels based on gender among the study participants. Plasma CXCL10 levels were significantly higher in males than in females (p<0.001, Figure 1B). There was a trend toward higher plasma levels of CXCL10 in male subjects in the non-CM and CM groups compared to female subjects in the non-CM or CM group (Figure 1C).

### Effect of −135G>A and −1447A>G promoter polymorphisms on plasma CXCL10 level

**Figure 2:**
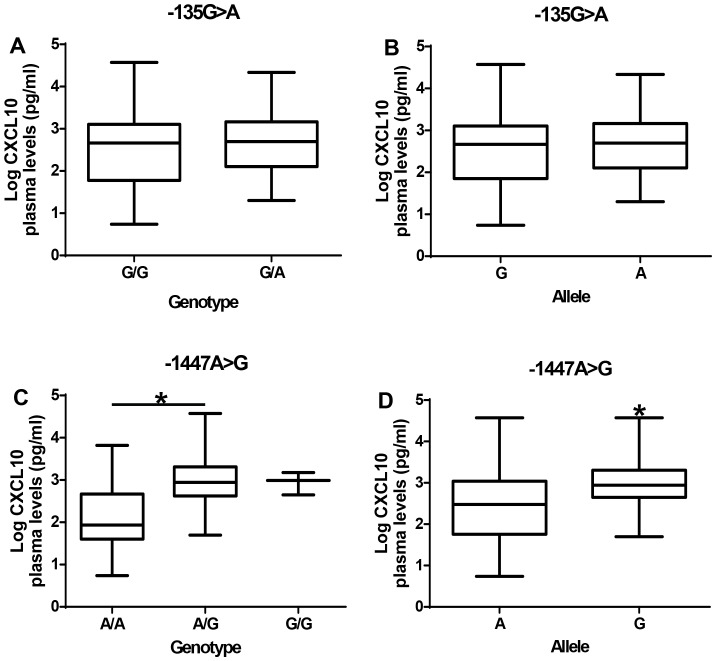
Effect of −135G>A and −1447A>G promoter polymorphisms on CXCL10 plasma levels. (A) Plasma CXCL10 levels among the genotype of −135G>A polymorphism. (B) Plasma CXCL10 levels among the allele of −135G>A polymorphism. (C) Plasma CXCL10 levels among the genotype of −1447A>G polymorphism. (D) Plasma CXCL10 levels among the allele of −1447A>G polymorphism. Box plot represent log-transformed medians with 25^th^ and 75^th^ percentiles, bars for 10^th^ and 90^th^ percentiles. Significant differences of CXCL10 median levels between the groups were determined by Mann-Whitney U test or Kruskal Wallis test with Dunn's multiple comparisons tests. Asterisk (*) indicates a significant difference (p<0.05).

There were no differences in plasma CXCL10 levels between −135(G/G) genotype and −135A allele carriers (Figure 2A and B). However, individuals with the −1447G allele had significantly higher plasma levels of CXCL10 than those with −1447(A/A) genotype (p<0.05; Figure 2C and D).

### Association between −135G>A and −1447A>G promoter polymorphisms with plasma CXCL10 levels stratified by disease group

**Figure 3:**
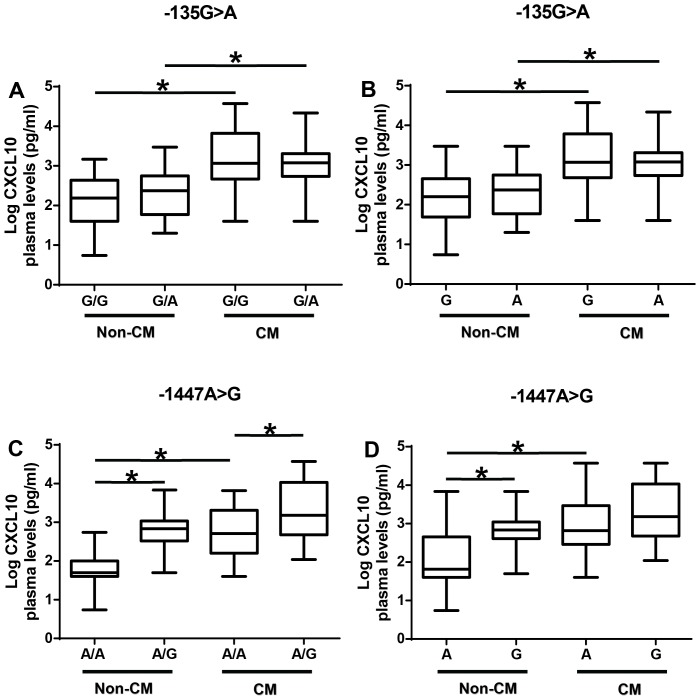
Association between −135G>A and −1447A>G polymorphism with plasma CXCL10 levels stratified by disease group. (A) Effect of −135G>A polymorphism genotype on plasma CXCL10 protein levels stratified by disease group. (B) Effect of −135G>A polymorphism allele on plasma CXCL10 protein levels stratified by disease group. (C) Effect of −1447A>G polymorphism genotype on plasma CXCL10 protein levels stratified by disease group. (D) Effect of −1447A>G polymorphism allele on plasma CXCL10 protein levels stratified by disease group. Box plot represent log-transformed medians with 25^th^ and 75^th^ percentiles, bars for 10^th^ and 90^th^ percentiles. The distribution of plasma CXCL10 expression levels among non-CM and CM patients segregated according to the genotype was analyzed with the Kruskal-Wallis test, followed by a Dunn's multiple-comparison, with p<0.05 considered significant. Asterisk (*) indicates a significant difference (p<0.05).

There were no differences between −135G>A genotypes in either the non-CM patients or the CM patients (Figure 3A). However, regardless of genotype, CM patients had significantly higher CXCL10 levels compared with non-CM patients (p<0.05). A similar pattern was observed with the alleles of −135G>A polymorphism (Figure 3B).

The non-CM patients with the −1447(A/G) genotype had significantly higher plasma CXCL10 levels than those with the −1447(A/A) genotype (p<0.05; Figure 3C). A similar pattern was observed in CM patients, however, CM patients had higher plasma levels of CXCL10 than non-CM patients (Figure 3C). Analyses of the alleles showed that non-CM and CM patients with −1447G alleles have higher plasma levels of CXCL10 than those with −1447A allele (p<0.05, Figure 3D).

### Association between −135G>A and −1447A>G promoter polymorphisms with plasma CXCL10 levels stratified by gender

**Figure 4:**
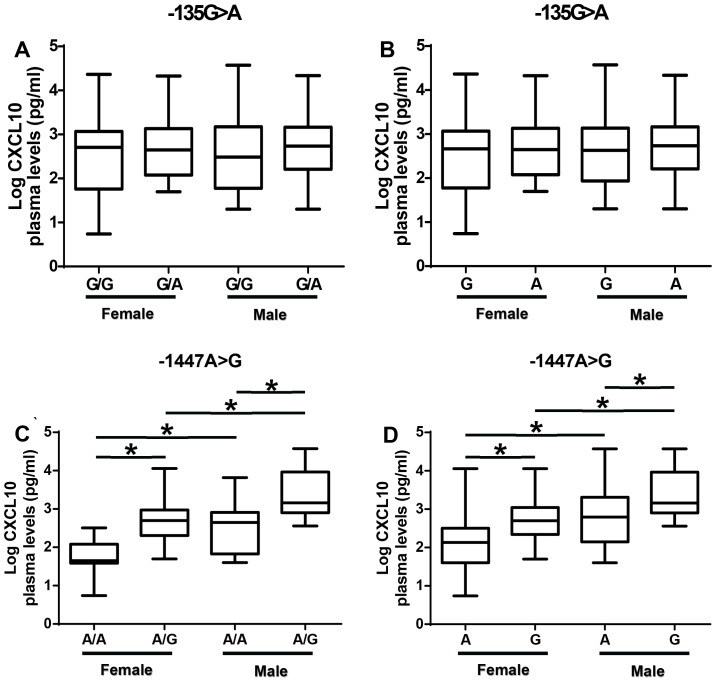
Association between −135G>A and −1447A>G polymorphism with plasma CXCL10 levels stratified by gender. (A) Effect of −135G>A polymorphism genotype on plasma CXCL10 protein levels stratified by gender. (B) Effect of −135G>A polymorphism allele on plasma CXCL10 protein levels stratified by gender. (C) Effect of −1447A>G polymorphism genotype on plasma CXCL10 protein levels stratified by gender. (D) Effect of −1447A>G polymorphism allele on plasma CXCL10 protein levels stratified by gender. Box plot represent log-transformed medians with 25^th^ and 75^th^ percentiles, bars for 10^th^ and 90^th^ percentiles. The distribution of plasma CXCL10 expression levels among females and males segregated according to the genotype was analyzed with the Kruskal-Wallis test, followed by a Dunn's multiple-comparison, with p<0.05 considered significant. Asterisk (*) indicates a significant difference (p<0.05).

There was no difference in CXCL10 plasma levels among females or males between −135(G/G) genotypes and −135A allele carriers (Figure 4A and B). Females with the −1447(A/G) genotype had significantly higher plasma levels of CXCL10 than those with −1447(A/A) genotype (p<0.05; Figure 4C). A similar pattern was observed with the males. However, males with −1447(A/A) or −1447(A/G) genotypes have higher plasma levels of CXCL10 than females with −1447(A/A) or −1447(A/G) genotypes respectively (p<0.05; Figure 4C). Analyses of the alleles showed that females and males with −1447G alleles have higher plasma levels of CXCL10 than those with −1447A allele (p<0.05, Figure 4D) suggesting a dominant effect of −1447G allele in expression of CXCL10.

## Discussion

Cerebral malaria accounts for approximately 80% of fatal malaria cases [2]. However, about 20% of malaria cases worldwide develop into CM suggesting that CM is a sub-population-specific targeted syndrome [3]–[5]. Although parasite diversity, host age, acquired immunity, and overall health status may influence the risk of developing CM, it has been estimated that 25% of this risk may be attributed to variations in host genes or genome [6], [23]. Human genetic background strongly influences susceptibility to malaria infection and subsequent progression to severe disease and death [6]. The geographic distribution of sickle cell disease, α-thalassemia, glucose-6-phosphate dehydrogenase deficiency, ovalocytosis, and the Duffy-negative blood group reinforces the principle that different populations have evolved different genetic variants to protect against malaria [6], [24].

Our previous studies indicate that the potent anti-angiogenic and apoptotic factor, CXCL10, is remarkably elevated in plasma/serum and cerebrospinal fluid of patients who died from CM compared to those who survive the disease after treatment confirming the association between elevated CXCL10 in CM patients with poor prognosis [15]–[17]. Additionally, CXCL10 was determined to be an independent chemokine predictor of fatal CM in Ghanaian children and Indians who died of CM [15]–[17]. These observations in human CM were confirmed in murine studies, which revealed that deletion of the CXCL10 gene partially protected mice against ECM when infected with *P. berghei* ANKA [12]. Campanella et al., [12] further observed that CXCL10 was the initial chemokine induced in the brain during the onset of murine ECM and was highly expressed in the brain of mice infected with *P. berghei* ANKA at the late stage of ECM. Interestingly, observations in both human and mouse models demonstrated CXCL10 as a strong indicator/predictor of CM in both human CM and murine ECM. In the present study, we assessed the association between polymorphisms in the CXCL10 gene promoter region and the clinical status of malaria patients and identified the genetic basis of plasma CXCL10 production in CM patients compared to non-CM patients.

Polymorphisms in the CXCL10 gene have been associated with diabetes [18], hepatitis [19], tuberculosis [20], Alzheimer's disease [21], and multiple sclerosis [22]. We investigated the association of CXCL10 polymorphism and risk of CM in an Indian cohort. The −1447A>G polymorphism in the promoter region of CXCL10 gene was associated with CM pathogenesis. The subjects in the present study carrying the G allele of the −1447A>G polymorphism were more likely to develop CM. However, previous studies in tuberculosis focusing on this single nucleotide polymorphism did not find any association with susceptibility to the disease [20]. The heterozygous −1447(A/G) genotype was found to be tightly associated with susceptibility to CM. It is likely that different SNPs of the same gene may have either an additive or an altered effect on the risk of developing CM. When CXCL10 promoter variant genotypes were combined together, the risk of CM increased and was associated with −135(G/G)/−1447(A/G) genotypes. Since, the −1447(A/G) genotype were found to be significantly associated with severe malaria, the −1447A>G substitutions seem to have an effect on the susceptibility to CM. However, the association was altered with −135(G/A)/−1447(A/G) genotypes suggesting that in the presence of −1447(A/G) genotype, the −135(G/A) genotype is dominant. Nevertheless, the significant association of −1447A>G substitution with CM as an individual SNP suggests that the −1447G allele is a putative risk allele for the development of CM.

In addition, there was a gender difference between the −1447A>G polymorphism and CM among the study participants. Male participants with the −1447(A/G) genotype were more susceptible to CM. However, males with −135(G/A) genotype were significantly protected from CM. The lack of a significant association in the female population may be due to the sample size used in this study [19]. Alternatively, studies in India indicate that about 76% of CM cases and 85% of CM-associated mortality occurs in males compared with females [25]. When the CXCL10 polymorphisms were combined, a significant association of −135(G/G)/ −1447(A/G) genotypes was found in males with CM. However, the association was altered with −135(G/A)/ −1447(A/G) genotypes suggesting that these promoter polymorphisms may be influenced by gender differences.

Regulation of the CXCL10 gene ultimately leads to differential expression of CXCL10 protein. We focused on the promoter region of CXCL10 gene since this region encompasses the regulatory machinery for gene expression and translation. We assessed the role of the polymorphisms in the CXCL10 promoter region in determining the plasma levels of CXCL10 and the genetic risk factors of CM in Indian patients. To our knowledge, this is the first report of an association between CXCL0 gene promoter polymorphism with the plasma levels in human CM, thereby providing supporting evidence regarding the functional relevance of CXCL10 in the clinical manifestation of CM.

The CXCL10 plasma levels in the subjects studied were highest in CM patients compared to non-CM patients. This observation was consistent with previous studies demonstrating an association between CXCL10 and fatal CM [15]–[17]. Furthermore, male participants had significantly higher plasma CXCL10 levels than female participants. Interestingly there was a trend toward higher CXCL10 plasma levels in male subjects with non-CM and CM compared to female subjects. Among humans, males are more susceptible to protozoan, fungal, bacterial, and viral infections than females and are more likely to die from these infections [26]–[28]. Surprisingly CXCL10 has been implicated in several diseases including CM [7], [12], [15]–[17], *Toxoplasma gondii* [29], tuberculosis [30]–[35], candidiasis [36], West Nile Virus [37], [38], hepatitis B virus [19], [39], and HIV [40] suggesting that CXCL10 may confer susceptibility to these infections.

The functional relevance of −135G>A and −1447A>G polymorphism was analyzed by assessing the association between the polymorphism and plasma CXCL10 levels in the patients. The presence of the SNP −135G>A in the CXCL10 promoter region does not seem to influence CXCL10 protein levels although CM patients have higher CXCL10 levels than non-CM. When plasma CXCL10 levels were analyzed according to −1447A>G polymorphism genotypes, heterozygous A/G and homozygous G/G genotypes were associated with plasma CXCL10 levels in the subjects. Treating each allele as a distinct group, it was determined that subjects with −1447G allele have high levels of CXCL10 suggesting a dominant effect of the G allele on expression of CXCL10. Furthermore, analysis of plasma CXCL10 levels according to −1447A>G polymorphism genotypes of CM or non-CM patients show increased levels of plasma CXCL10 in either CM or non-CM patients with −1447(A/G) genotype. Males with −1447(A/G) genotypes have higher CXCL10 levels than females with the same genotype supporting the evidence that there may be gender difference in CXCL10 production.

CXCL10 mediates leukocyte trafficking and modulates innate and adaptive immune responses [41]. Secretion of CXCL10 from leukocytes, neutrophils, eosinophils, monocytes, epithelia, keratinocytes, endothelial, and stromal cells plays an important role in inflammatory responses to IFN-γ [11], [42], [43]. CXCL10 up-regulates the production of Th1 cytokines and down-regulates the production of Th2 cytokines [41]. CXCL10 induces a robust up-regulation of inflammatory reactions characterized by the production of IFN-γ, thus exerting important Th1 cell protective activity against infections sustained by intracellular bacteria, parasites, and viruses [41]. Elevated levels of CXCL10 may contribute to vascular injury resulting in the dysfunction of the blood-brain barrier and leukocyte trafficking and accumulation resulting in local hyper-inflammation [7], [16]. Furthermore, excess production of CXCL10 has been shown to be deleterious to brain cells suggesting that increased production in CM patients may contribute to the neuro-pathogenesis and blood-brain barrier damage associated with CM [7]. Interestingly, it has recently been demonstrated that pharmacologically reducing or eliminating CXCL10 improved survival of mice with experimental cerebral malaria [7], [12].

Despite the novelty and importance of our findings, our study may have limitations that need to be addressed. First, this was a hospital-based case control study, therefore selection bias was unavoidable and the participants may not be fully representative of the general population. Second, a single case-control study is not sufficient to fully interpret the relationship between CXCL10 polymorphism and susceptibility to CM. It will be interesting to clarify the exact association between the CXCL10 gene and susceptibility to CM in various populations. Finally, the polymorphisms investigated, based on their functional considerations, might not offer a comprehensive view of the genetic variability of CXCL10 gene. Further mapping studies will be required to establish the molecular relevance of these findings.

In conclusion, the present study has provided strong evidence that the CXCL10 gene promoter −1447A>G polymorphism is a marker of susceptibility to CM in individuals living in malaria endemic areas such as India. Additionally, the −1447A>G polymorphism in the CXCL10 gene promoter region is linked to increased plasma CXCL10 production. Furthermore, there were gender differences in CM susceptibility and production of plasma CXCL10 with males being discriminately affected. We have identified an association between the −135(G/A) genotype and protection from CM among males. Taken together, these findings indicate an association between CXCL10 genotype and high CXCL10 production during CM, which eventually plays a role in severity of the disease. Since the functionality of these polymorphisms are not clearly known, the association that we have found between the CXCL10 promoter polymorphism genotypes and CM may be due to differential expression of CXCL10 affecting clinical outcomes of malaria. Collectively, our results suggest a hitherto unrecognized role for CXCL10 polymorphisms and gender in CM pathogenesis. A better understanding of gender disparities may ultimately lead to new pharmacological targets for the treatment of CM. Further studies are needed in other malaria endemic populations to confirm or replicate these findings and investigate exactly how these CXCL10 promoter variants protect or render susceptibility against CM.

## Methods

### Study sites

The study was conducted in a malaria endemic region in Madhya Pradesh, India, which accounts for 23% of all malaria cases in the state [44]. The study samples were obtained from two sites: Nethaji Subash Chandra Bose Hospital (a regional referral hospital) in Jabalpur and Civil Hospital (a primary hospital) in Maihar, Satna District. Both *P. vivax* and *P. falciparum* are prevalent in this area, and *P. falciparum* transmission occurs primarily during the monsoon and post-monsoon seasons (July - January). Previous studies revealed that malaria is present in all age groups, with the highest prevalence occurring in children between 8–14 years of age [45], [46].

### Study subjects

All subjects were enrolled after written informed consent in the native Hindi language was obtained from patients or guardians of patients with unarousable coma. Informed consent and human subject research guidelines of the National Institutes of Health (NIH, USA), and the Centers for Disease Control and Prevention (CDC) in the United States were followed. The study was approved by the IRB committees of the Morehouse School of Medicine (USA) and the National Institute of Malaria Research (India). Pregnant women and patients with other severe disease such as respiratory distress without CM and non-CM related coma, were excluded from the study. Data relating to age, sex, and level of parasitemia were obtained from medical records.

### Enrollment criteria

#### Cerebral malaria

All CM patients fulfilled the World Health Organization's (WHO) definition of CM [47], and had Glasgow coma score of ≤8, a *P. falciparum* parasitemia, and no other clinically evident cause of impaired consciousness [48].

#### Non-cerebral malaria

Malaria patients who had fever with *P. falciparum* parasitemia of <25,000 parasites/µl of blood (detected microscopically from blood smears) and no evidence of impaired consciousness, seizures, and no past history of mental illness, meningitis, or accidental head injury were included in this group.

Relevant clinical data and information were recorded for each patient from physician's records [49]. Venous blood samples from children (2–5 ml) and adults (10 ml) were collected soon after enrollment into the study and prior to commencement of anti-malarial treatment or transfusions. A total number of 66 CM and 69 non-CM patients were recruited into the study.

### DNA isolation and genotyping

Human genomic DNA from patients were purified from whole blood using QIAamp DNA Blood Mini Kit (Qiagen, Valencia, CA) according to the manufacturer's instruction. After isolation and purification, the DNA was quantified on a NanoDrop 2000 (Thermo Scientific, Rockford, IL) to measure concentration and assess the purity of the DNA through standard A_260/_A_280_ and A_260/_A_230_ ratios. The DNA was diluted to 400 µl total, except where yields were lower. Three microliters (3 µl) aliquot of the DNA solution was evaluated for DNA length distribution and potential degradation by electrophoresis on 1% agarose gel against a molecular weight ladder with ethidium bromide staining. In the absence of reported data correlating CXCL10 polymorphisms with CM, we performed extensive literature search (PubMed and Scopus) on reported SNPs in the promoter region of the CXCL10 gene. We used dbSNP database available at National Center for Biotechnology Information (NCBI) web site (http://www.ncbi.nlm.nih.gov/projects/SNP) to determine the exact nucleotide sequence and frequencies of each SNP. GeneView was used to determine the alignment of the SNPs on the RefSeq sequence. We performed analyses on polymorphisms in the promoter region of CXCL10 gene that were previously associated with tuberculosis (1447A>G [rs4508917], 135G>A [rs56061981], and 872G>A [rs4256246]) [20] and hepatitis B (1596C>T [rs74810361]) [19] using polymerase chain reaction-restriction fragment length polymorphism (PCR-RFLP) and appropriate primers and restriction enzymes (Table 7). However, only two SNPs in the CXCL10 promoter region, −135G>A (rs56061981) and −1447A>G (rs4508917) [20], were identified in the Indian cohorts. We concentrated our analysis on these two SNPs in this study.

**Table 7:** List of Promoter Single Nucleotide Polymorphisms Screened.

Name	SNP ID	Primers	Annealing Temperature (°C)	Product	Enzyme	Restriction Fragment
−1447A>G	rs4508917	5′-TTGGTCAGGGAATGGAAAAG-3′	55	290 bp	*SacI*	145/145 bp
		5′-CGGTTTCCCACAGCTAATTC-3′				
−135G>A	rs56061981	5′-CCGTTCATGTTTTGGAAAGTGA-3′	48	123 bp	*BstBI*	61/62 bp
		5′-GGGAAGTCCCATGTTGCAGATT-3′				
−872G>A	rs4256246	5′-TGAAATTAAGTTTTGCCACGA-3′	52	155 bp	*NcoI*	22/133 bp
		5′-GGAAACAGGTTGATTTACCATG-3′				
−1596C>T	rs74810361	5′-GCAGATACTGTCTCAGAACCTGGTA-3′	57	499 bp	*XbaI*	174/325 bp
		5′-TGTCACCATCTCTCATTTTGATTGT-3′				

SNP  =  single nucleotide polymorphism; ID  =  identification; bp  =  base pairs

Genotyping of −135G>A and −1447A>G was performed by PCR-RFLP and the PCR was performed in a 25 µl reaction comprising 0.5 µM of each primer pair and Phusion Flash High-Fidelity PCR Master Mix (Thermo Scientific, Rockford, IL).

Amplification of the specific DNA fragments were performed using Multigene Gradient Thermal cycler (Labnet International, Inc.) according to the following conditions: the reaction was initially heated at 98°C for 10 seconds and DNA amplification was achieved by 30 cycles of 98°C for 1 second, annealing for 5 seconds and extension at 72°C for 15 seconds/1 kb. The final elongation step was 72°C for 1 minute. Ten microliters (10 µl) of the PCR product was digested by the required enzyme in the presence of the accompanying buffer in a final volume of 20 µl incubated at the temperature with optimal activity of the enzyme overnight.

The SNP at the −135 position in the promoter with a change in G>A was analyzed by amplification of the 123-bp size fragments by PCR using primers CCGTTCATGTTTTGGAAAGTGA and GGGAAGTCCCATGTTGCAGATT at annealing temperature of 48°C. The final amplicon, consisting of a restriction site for *BstBI* in the presence of G allele was identified by 61-bp and 62-bp products detected using gel electrophoresis. The SNP at position −1447 in the promoter with an A>G change was analyzed by amplifying the 290-bp fragment using the primers TTGGTCAGGGAATGGAAAAG and CGGTTTCCCACAGCTAATTC at an annealing temperature of 55°C to generate a restriction site for the *SacI* enzyme in the presence of the G allele. The mutant PCR product was cleaved to produce a fragment of 145-bp while the wild-type remained uncut.

### Functional analysis in vivo

To determine the association between CXCL10 polymorphism and plasma CXCL10 levels, we examined plasma CXCL10 levels in patients with specific SNPs using a commercially available Human CXCL10/IP-10 Quantikine ELISA kit (R&D Systems, Minneapolis, MN). CXCL10 levels were measured using optimal concentrations of standards and antibodies according to the manufacturer's instructions. The data was analyzed at 450 nm wavelength using Spectra Max 190 fluorescence microplate reader (Molecular Devices Corp., Sunnyvale, CA).

### Statistical analysis

Genotype and allele frequency were calculated by direct counting. Chi square tests were used to examine the differences in allele frequencies and genotype distribution between non-CM and CM patients. Fisher's exact test was used when cell count was <5. Multivariable logistic regression analysis was performed to adjust risk factors such as age, gender, and parasitemia. The association between genotyped polymorphisms and risk of CM was estimated by p values, odds ratio (OR) and 95% confidence intervals (CI). The deviation of the genotypic frequencies from Hardy-Weinberg equilibrium and linkage disequilibrium SNPs in the promoter region of CXCL10 gene sequence were examined using Haploview software [50]. The association of genotype and plasma CXCL10 were analyzed by Mann-Whitney U test or Kruskal-Wallis test followed by a Dunn's multiple-comparison. A p value of <0.05 was considered significant. SigmaPlot (version 11.0) with SigmaStat (version 3.5) integration (Chicago, IL) and SAS version 9.2 (Cary, NC) software for windows were used for statistical analysis. GraphPad Prism version 6 (La Jolla, CA) for windows was used to generate all the graphs. The power of the study was calculated by using online tool Quanto (version 1.2.4) with the following parameters: a dominant model, a population risk of 2% for CM, a minor allele frequency of 20% for OR  = 3.0 at 2-sided p = 0.05 for sample numbers 64 for CM and 64 non-CM to achieve 86% power when the OR of 3.0 was returned.
